# A First-Principles Study of Formaldehyde Adsorption on the Surface of ZnO $\left[20\bar{2}1\right]$ High Index Polar Facet

**DOI:** 10.3390/ma19122661

**Published:** 2026-06-20

**Authors:** Chao Ma, Jingze Yao, Xuefeng Xiao, Yujie He, Hao Zhang

**Affiliations:** 1 School of Electrical and Information Engineering, North Minzu University, Yinchuan 750021, China; nexusyao48@gmail.com (J.Y.); xuefengxiao@nmu.edu.cn (X.X.); heyujie2001@outlook.com (Y.H.); 1804894618z@gmail.com (H.Z.); 2 Institute of Semiconductor Crystals and Ceramic Materials, Helanshan Laboratory, Yinchuan 750021, China; 3 Key Laboratory of Physics and Photoelectric Information Functional Materials, North Minzu University, Yinchuan 750021, China; 4 Microelectronics and Solid-State Electronics Device Research Center, North Minzu University, Yinchuan 750021, China

**Keywords:** formaldehyde, zinc oxide, gas-sensitive mechanism, surface adsorption, density functional theory

## Abstract

High-sensitivity detection of formaldehyde is critically important for environmental protection and public health. Zinc oxide (ZnO) is a widely used core material for chemiresistive gas sensors; however, its conventional low-index facets suffer from a limited number of active sites, creating a bottleneck for further sensitivity enhancement. To overcome this limitation, this study pioneers the application of the highly reactive ZnO $\left[20\bar{2}1\right]$ high-index polar surface for formaldehyde detection. By leveraging its unique stepped atomic configuration and unprecedented density of coordination-unsaturated active sites, we systematically investigate the formaldehyde adsorption behavior and the underlying sensing mechanism using first-principles calculations based on density functional theory (DFT). The pristine ZnO $\left[20\bar{2}1\right]$ surface exhibits intrinsic metallic character. At a coverage of 1 monolayer (ML), the most stable G1 configuration achieves an adsorption energy of −1.54 eV per CH_2_O molecule. Within a 2 × 1 supercell, formaldehyde adopts both associative and dissociative adsorption modes. At a lower coverage, formaldehyde forms a stable bidentate structure through dual C–O and Zn–O bonding interactions. Electronic structure analysis reveals significant orbital hybridization and interfacial charge redistribution upon adsorption. Notably, associative adsorption opens a bandgap of 0.04 eV at the Fermi level, inducing a metal-to-semiconductor transition. In contrast, dissociative adsorption results in pronounced n-type doping, thereby elucidating the microscopic origin of the resistivity decrease observed in ZnO-based sensors. Overall, this work highlights the structural advantages of high-index facets and demonstrates for the first time the superior formaldehyde adsorption capability of the ZnO $\left[20\bar{2}1\right]$ facet, providing robust theoretical guidance for the rational design of next-generation, high-performance gas-sensing materials.

## 1. Introduction

Formaldehyde (CH_2_O) is a typical volatile and toxic gas that is widely present in processes such as industrial emissions [1], agricultural production [2], and chemical synthesis [3], posing a serious threat to public health and safety. The primary health hazard of CH_2_O lies in its well-established carcinogenicity; it can interact with human cells at the DNA level and induce carcinogenesis [4]. Studies by Awan et al. [5] have identified oral and pharyngeal cancers as representative malignancies associated with exogenous CH_2_O exposure. In addition, research by Asif Ahmad demonstrates that CH_2_O exposure levels are closely correlated with the incidence and severity of various respiratory diseases, which, in severe cases, may compromise cardiopulmonary function and become life-threatening [6]. Given these risks, the development of high-sensitivity, on-site, and rapid detection methods for CH_2_O has become an urgent priority in environmental monitoring and public health protection [7]. Conventional detection techniques, including gas chromatography–mass spectrometry (GC–MS) [8] and high-performance liquid chromatography (HPLC) [9], are limited in practical, on-site applications due to their high cost, bulky instrumentation, and poor portability. In recent years, driven by the growing demand for rapid CH_2_O detection, semiconductor gas sensors have attracted extensive research interest and have been increasingly adopted in industrial applications [10]. Compared with traditional large-scale analytical instruments, these sensors offer significant advantages, including excellent portability, fast response, and the potential for ultra-high sensitivity through bandgap engineering of sensing materials, as well as simple device structures and low manufacturing costs [11]. Consequently, current research on gas sensors has increasingly focused on the development and physicochemical property tuning of high-performance sensing materials [12]. Among these, metal oxide semiconductors have emerged as the most widely used class of sensing materials due to their strong gas-response characteristics, robust environmental stability, and well-established fabrication processes [13].

ZnO is a typical wide-bandgap metal oxide semiconductor, characterized by a high exciton binding energy [14] and a bandgap of up to 3.37 eV [15], making it one of the most widely utilized core sensing materials in gas-sensing applications. At the microscopic scale, ZnO nanomaterials exhibit a large specific surface area and abundant lattice defects, which significantly increase the density of effective surface active sites. This, in turn, enhances adsorption capacity and surface coverage at the macroscopic level [16], thereby endowing ZnO with strong potential for detecting volatile hazardous gases such as CH_2_O. To date, studies on the gas-sensing properties of ZnO surface structures have primarily focused on easily synthesized non-polar surfaces and low-index polar surfaces. While experimental investigations provide crucial macroscopic metrics for gas sensor performance [17], characterizing the atomic-level interactions and transient charge-transfer dynamics at the gas–solid interface remains profoundly challenging. In this regard, first-principles calculations based on density functional theory (DFT) [18] have emerged as an indispensable and powerful tool in modern materials science [19]. By systematically evaluating parameters such as adsorption energy [20], charge density difference [21], and partial density of states (PDOS) [22], DFT studies provide profound mechanistic insights into target gas recognition, molecular dissociation, and the resulting conductivity modulation. Consequently, a rapidly growing body of theoretical literature has successfully utilized DFT to elucidate the role of specific surface facets, decode complex interfacial redox reactions, thereby guiding experimental efforts from trial-and-error paradigms toward predictive design [23]. For instance, in investigations of the ZnO polar [0001] surface, Dazhi Chen et al. [24] systematically elucidated the adsorption behavior and sensing mechanism of CH_2_O molecules. Similarly, in studies of the ZnO $\left[10\bar{1}0\right]$ low-index non-polar surface, Wentao Jin et al. [25] identified a stable chain-like adsorption configuration for CH_2_O and demonstrated that electrostatic interactions induced by molecular polarization play a dominant role in governing the geometric stability and energy evolution of the adsorption system. Furthermore, Lv et al. [26] examined the adsorption behavior of SF_6_ molecules on ZnO [0001] and $\left[10\bar{1}0\right]$ surfaces, revealing that oxygen vacancy defects significantly facilitate molecular activation and bond dissociation. They also showed that strong hybridization between F-2p and Zn-3d orbitals is the fundamental origin of the enhanced adsorption energy.

Although the excellent gas adsorption performance of ZnO low-index crystal planes for molecules such as CH_2_O has been widely demonstrated, further enhancement of their sensing performance is hindered by the limited density of active sites on these relatively flat surfaces. Consequently, recent research has increasingly shifted toward high-index polar crystal planes with intrinsically higher surface activity. These planes possess abundant step sites and undercoordinated corner atoms, resulting in enhanced chemical reactivity and a greater density of effective adsorption sites. Theoretically, this structural complexity is expected to confer stronger CH_2_O capture capability, suggesting that the gas-sensing performance of high-index polar surfaces should surpass that of conventional low-index non-polar surfaces. Growing experimental and theoretical evidence supports this hypothesis. For instance, Weile Jiang et al. [27] compared the adsorption behavior of various gases on ZnO [0001], $\left[10\bar{1}0\right]$, and $\left[10\bar{1}1\right]$ surfaces, and found that all tested gas molecules exhibited the highest adsorption energies on the $\left[10\bar{1}1\right]$ high-index polar surface. Similarly, Li et al. [28] investigated the catalytic decomposition mechanism of ammonium perchlorate on the ZnO $[2\bar{11}0]$ high-index polar surface, revealing that lattice oxygen on this surface can react with the thermal decomposition intermediate NH_3_ to generate oxygen vacancies. This process significantly enhances catalytic activity, far exceeding that observed on low-index surfaces. In summary, existing studies have clearly demonstrated the considerable potential of ZnO high-index polar crystal planes in gas sensing and catalytic applications.

With the rapid development of controllable ZnO crystal growth techniques, the stable and controllable synthesis of high-index polar crystal planes has gradually been achieved, providing an experimental foundation for their device-level applications [29]. In previous work, our group found that under specific chemical potential conditions, the surface energy of the ZnO $\left[20\bar{2}1\right]\;$ high-index polar crystal plane can be lower than that of conventional low-index surfaces [30]. According to the Wulff construction principle of crystal growth [31], this result provides a theoretical basis for the controllable synthesis and stabilization of the ZnO $\left[20\bar{2}1\right]$ crystal plane [32]. From a structural perspective, the ZnO $\left[20\bar{2}1\right]$ surface exhibits a stepped, alternating atomic arrangement. Its unit cell contains three coordination-unsaturated Zn active sites with dangling bonds, providing a favorable structural basis for the efficient adsorption and activation of CH_2_O molecules. This makes it a highly promising candidate for high-performance CH_2_O gas-sensing applications. At present, several experimental studies have successfully achieved the controlled fabrication and functional application of the ZnO $\left[20\bar{2}1\right]$ crystal plane, further confirming its feasibility for practical engineering applications. For example, Salim et al. [33] prepared $\left[20\bar{2}1\right]$ -oriented ZnO thin films on lithium niobate (LiNbO_3_) substrates via RF sputtering and performed detailed structural characterization. Chiu et al. [34] synthesized Ga-doped ZnO single crystals on silicon substrates using chemical vapor deposition (CVD) and demonstrated that Ga incorporation effectively reduces the surface energy of high-index crystal planes, enabling stable growth of the ZnO $\left[20\bar{2}1\right]$ surface. Moreover, field emission devices based on this crystal plane exhibited an 18-fold increase in the field emission enhancement factor (β) compared with conventional surfaces. Zhang et al. [35] fabricated ZnO $\left[20\bar{2}1\right]$ thin films via magnetron sputtering and showed that their preferential growth behavior could be effectively tuned by adjusting the Al doping concentration. In addition, Sheshamani et al. [36] performed 1.2 MeV proton irradiation on different ZnO crystal planes and found that only the ZnO $\left[20\bar{2}1\right]$ surface was able to generate hydroxyl groups and form Zn–OH composite structures. This result confirms that surface strain in this crystal plane can effectively modulate the electronic structure and physicochemical properties of the material.

In summary, the ZnO $\left[20\bar{2}1\right]$ high-index polar crystal plane has been successfully explored in the fields of controllable synthesis, catalytic reactions, and electronic devices, and its application in CH_2_O gas sensing is supported by substantial theoretical and experimental evidence. However, current research on the adsorption behavior, electronic interaction mechanisms, and gas-sensing response characteristics of CH_2_O molecules on the ZnO $\left[20\bar{2}1\right]$ surface remains relatively limited, and systematic theoretical investigations are urgently needed to clarify its performance potential and underlying sensing mechanisms. Motivated by this, the present work employs first-principles calculations based on density functional theory (DFT) to systematically investigate the adsorption behavior and microscopic interaction mechanisms of CH_2_O molecules on the ZnO $\left[20\bar{2}1\right]$ surface. First, various possible adsorption sites are constructed across different supercell models, and the most stable adsorption configurations are identified through adsorption energy calculations combined with geometric structural analysis. Second, the electronic properties, including band structure, partial density of states (PDOS), and charge density difference, are systematically examined to elucidate the orbital hybridization and charge transfer behavior between CH_2_O molecules and surface active sites at the atomic scale. The results of this study aim to clarify the intrinsic performance advantages and microscopic sensing mechanisms of the ZnO $\left[20\bar{2}1\right]$ high-index polar crystal plane toward CH_2_O detection, thereby providing a solid theoretical foundation and valuable data support for the rational design and optimization of high-performance ZnO-based formaldehyde gas sensors.

## 2. Computation Methods

All calculations in this work are based on DFT and implemented using the Vienna Ab initio Simulation Package (VASP, version 6.1.0). The interaction between core and valence electrons is described using the projector augmented wave (PAW) method [37], while the exchange–correlation functional is treated within the generalized gradient approximation (GGA) using the Perdew–Burke–Ernzerhof (PBE) form [38]. Given that the adsorbate-substrate interactions in this system are strictly dominated by strong chemisorption, van der Waals (vdW) corrections were not included in the present calculations. A plane-wave basis set with a kinetic energy cutoff of 500 eV is employed. Brillouin zone integrations are performed using a 2 × 4 × 1 Monkhorst–Pack k-point mesh. During structural optimization, the conjugate gradient algorithm is used to fully relax atomic positions, while the unit cell volume and lattice parameters are kept fixed. The electronic self-consistent loop is converged to an energy threshold of 1 × 10^−8^ eV, and structural relaxation is considered complete when the Hellmann–Feynman forces on all atoms are less than 0.02 eV/Å. To ensure high-precision electronic minimization, projection operators are evaluated in reciprocal space to account for the non-spherical charge density distribution within the PAW spheres and to reduce grid-related numerical errors. Electronic partial occupancies are treated using the Gaussian smearing method with a smearing width of 0.05 eV. Post-processing analyses, including band structure and density of states (DOS) calculations, are performed using the VASPKIT package (version 1.5) [39].

For adsorption energy, the adsorption energy of a single CH_2_O molecule is expressed by the following formula:

$$E_{\mathrm{ads}}\;=\;\frac{1}{N}\left(E_{\mathrm{total}}-E_{\mathrm{slab}}-NE_{\mathrm{gas}}\right)$$

where $E_{\mathrm{total}}$ is the total energy of the adsorption system after, $E_{\mathrm{slab}}$ is the total energy of the pristine $\left[20\bar{2}1\right]$ surface without CH_2_O molecules, and $E_{\mathrm{gas}}$ is the energy of an isolated CH_2_O molecule, and *N* denotes the number of CH_2_O molecules involved in the adsorption process.

The charge density difference (CDD) before and after adsorption is expressed by the following equation:

$$\Delta \rho \;=\;\rho_{\mathrm{total}}-\rho_{\mathrm{slab}}-\rho_{\mathrm{gas}}$$

where $\rho_{\mathrm{total}}$ is the charge density of the total system, while $\rho_{\mathrm{slab}}$ and $\rho_{\mathrm{gas}}$ represent the charge density of the pure ZnO [$20\bar{2}1$] surface without adsorbed CH_2_O and the charge density of CH_2_O, respectively.

**Figure 1:**
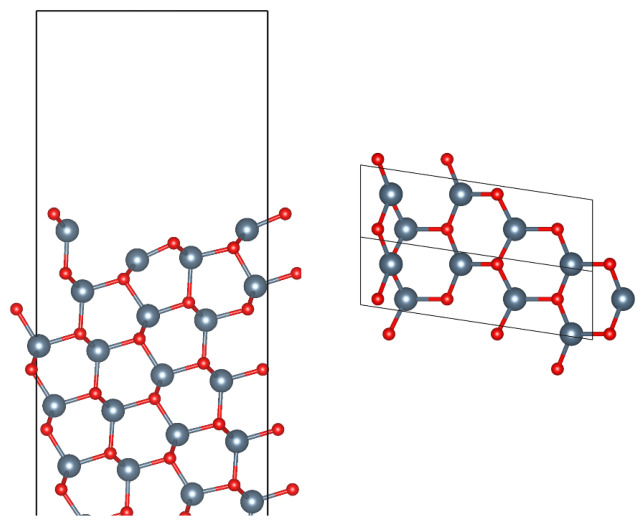
ZnO [$20\bar{2}1$] slab model, side view (**left**), top view (**right**). Gray spheres represent Zn atoms and red spheres represent O atoms. The same color scheme is adopted for all subsequent structural figures.

The ZnO $\left[20\bar{2}1\right]$ high-index polar crystal plane slab model constructed in this study is shown in Figure 1. Considering that gas adsorption coverage can significantly influence the surface electronic structure and reactivity of materials [40], two computational models were established to systematically investigate CH_2_O adsorption behavior under different coverages: a 1 × 1 primitive supercell and a 2 × 1 supercell extended along the x-axis. The 1 × 1 supercell contains 24 Zn atoms and 23 O atoms, with optimized lattice parameters of 10.91 Å × 3.25 Å × 35.69 Å. The 2 × 1 supercell contains 48 Zn atoms and 46 O atoms, with lattice parameters of 10.91 Å × 6.50 Å × 35.69 Å. Structural analysis shows that the top surface of the 1 × 1 supercell contains three coordination-unsaturated Zn atoms with single dangling bonds, as well as one Zn atom in a fully coordinated environment without dangling bonds. After full geometric relaxation, the O–Zn–O bond angle near the surface hollow site adjusts to 103.31°, accompanied by significant surface reconstruction that leads to a stepped atomic configuration with enhanced reactivity. The ZnO $\left[20\bar{2}1\right]$ surface exhibits unique crystallographic characteristics, where Zn and O atoms are arranged in alternating step-like motifs. From a side view, this surface can be regarded as a periodic stacking of ZnO $\left[10\bar{1}0\right]$ non-polar planes. When viewed along the $\left[20\bar{2}1\right]$ direction, the characteristic hexagonal ring structure of wurtzite ZnO becomes clearly visible. Structurally, this high-index polar surface can be interpreted as a composite of the $\left[10\bar{1}0\right]$ non-polar plane and the [0001] polar plane. In particular, regions along the y-axis of the surface form a stepped architecture derived from the stacking of $\left[10\bar{1}0\right]$ planes. This structure provides a high density of coordination-unsaturated active sites, which serve as the primary adsorption and activation centers for CH_2_O molecules and constitute the key focus of the subsequent analysis in this work.

**Figure 2:**
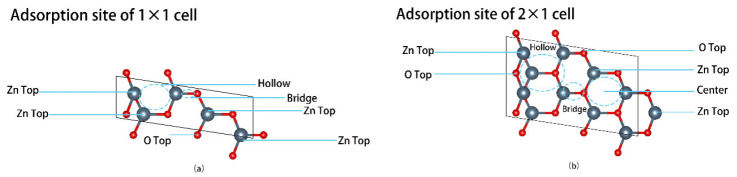
Top view of adsorption sites on the surface of ZnO [$20\bar{2}1$] facet; (**a**) shows a 1 × 1 unit cell, and (**b**) shows a 2 × 1 supercell.

Figure 2 illustrates the distribution of candidate adsorption sites on the ZnO $\left[20\bar{2}1\right]$ high-index polar surface under different supercell configurations. Geometric analysis shows that, after constructing the 2 × 1 supercell via extension along the x-axis, the surface atomic arrangement and intrinsic structural characteristics remain essentially unchanged, representing a periodic repetition of the 1 × 1 unit cell. This confirms the structural stability and representativeness of the selected supercell model. Based on these structural features, five representative adsorption sites were systematically selected in both supercells: Zn top site, O top site, bridge site, hollow site, and center site, thereby covering all possible CH_2_O adsorption configurations on this surface. In particular, due to the inherent stepped atomic structure of ZnO $\left[20\bar{2}1\right]$, four coordination-unsaturated Zn atoms are exposed at the step edge region along the y-axis. The unique steric environment and localized charge distribution in this region provide abundant high-activity adsorption sites, which are expected to play a key role in subsequent CH_2_O adsorption processes.

## 3. Results and Discussion

### 3.1. Adsorption Configuration at 1 ML Coverage

**Figure 3:**
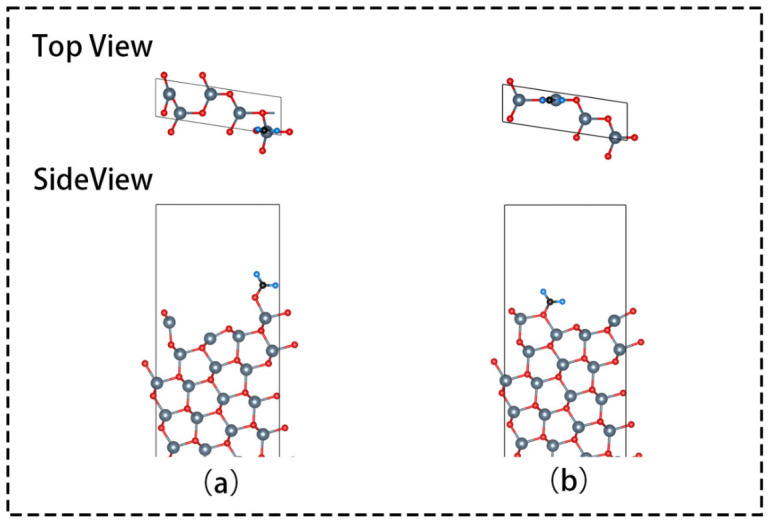
Two stable adsorption configurations, G1 (**a**) and G2 (**b**), on the ZnO [$20\bar{2}1$] facet at 1 ML coverage. Black spheres represent C atoms, and blue spheres represent H atoms. The same color scheme is adopted for all subsequent structural figures.

For the 1 × 1 primitive supercell, corresponding to single-molecule adsorption coverage (one CH_2_O molecule per unit cell), full structural relaxation was performed for all initial candidate adsorption configurations. Two energetically stable adsorption structures, denoted G1 and G2, were ultimately obtained. The optimized geometries are presented in Figure 3. Among them, configuration G1 is the most stable adsorption structure, with an adsorption energy of −1.54 eV per CH_2_O molecule, as shown in Figure 3a (side and top views). Structural analysis indicates that the CH_2_O molecule retains its intrinsic planar geometry after adsorption, with no significant molecular deformation. The oxygen atom of CH_2_O forms a stable Zn–O bond with a coordination-unsaturated Zn atom located at the surface step edge along the c-axis direction. Meanwhile, the hydrogen atom experiences electrostatic attraction from a nearby surface oxygen atom, resulting in a slight displacement toward the surface. The surface atomic framework shows no obvious reconstruction before and after adsorption, indicating that the overall structure remains stable. The metastable configuration G2, shown in Figure 3b, has an adsorption energy of −1.45 eV per CH_2_O molecule. This configuration corresponds to a typical bridge-site adsorption mode, in which the CH_2_O molecule maintains its planar structure while adsorbing at the step-edge active region and interacting with two coordination-unsaturated Zn atoms. The oxygen atom of CH_2_O simultaneously bonds with both Zn atoms, effectively saturating their dangling bonds and occupying the surface vacancy at the step site. From the crystallographic perspective, the CH_2_O molecule is oriented approximately perpendicular to the ZnO $\left[20\bar{2}1\right]$ surface and aligned along the [0001] direction. The two initially separated Zn atoms at the step edge become indirectly connected through the bridging oxygen atom of CH_2_O, forming a stable Zn–O–Zn linkage. Bond analysis shows that the two newly formed Zn–O bond lengths are 2.07 Å and 2.11 Å, respectively, while the Zn–O–Zn bond angle is 113.03°, which is close to typical Zn–O bond geometries in bulk ZnO. This further confirms the structural stability of the bridge-type adsorption configuration.

**Figure 4:**
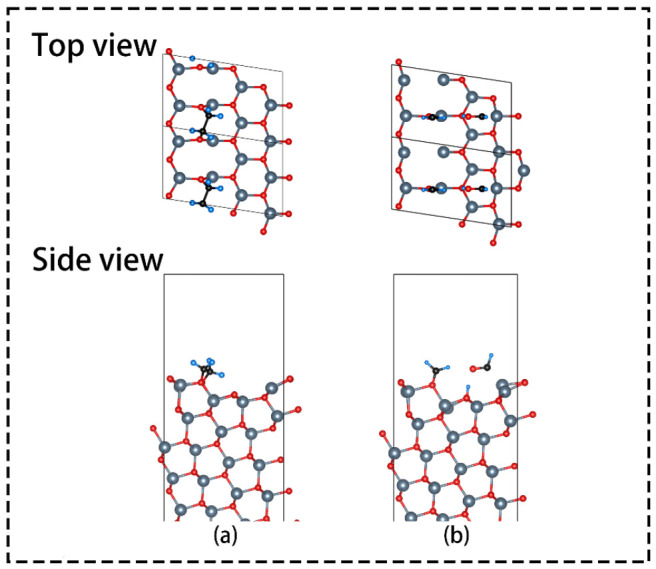
Two adsorption configurations on the ZnO $\left[20\bar{2}1\right]$ facet at 2 × 1 unit cell coverage: (**a**) the H1 configuration of covalent adsorption, and (**b**) the H2 configuration of dissociative adsorption.

At a 1 ML single-layer coverage consistent with the 1 × 1 supercell, a 2 × 1 supercell model containing two CH_2_O molecules was constructed. After full structural relaxation of all initial adsorption configurations, two energetically stable double-molecule adsorption structures, denoted H1 and H2, were obtained. The optimized geometries are shown in Figure 4. Configuration H1, shown in Figure 4a, corresponds to an associative co-adsorption configuration, with an average adsorption energy of −1.27 eV per CH_2_O molecule. Structural analysis indicates that the two CH_2_O molecules form a C–C covalent bond, resulting in dimer formation. The original sp^2^ hybridization of the carbon atoms is completely disrupted, with the bonding configuration transitioning toward an sp^3^-like tetrahedral geometry, indicating strong chemisorption. The terminal oxygen atoms of the dimer simultaneously form Zn–O coordination bonds with two coordination-unsaturated Zn atoms located at the surface step. Meanwhile, the C–H bonds bend noticeably toward the surface normal direction. Quantitative bond analysis shows that the two Zn–O bond lengths are 2.15 Å and 2.09 Å, respectively, with a corresponding Zn–O–Zn bond angle of 115.18°. Notably, the stepped surface morphology is significantly altered under this cooperative adsorption: the two undercoordinated Zn atoms originally located at the step edge become bridged via the CH_2_O -derived oxygen atoms, forming a stable Zn–O–Zn linkage. This process induces pronounced cooperative relaxation of the surface atomic structure. Configuration H2, shown in Figure 4b, is a mixed dissociative adsorption configuration with an average adsorption energy of −1.07 eV per CH_2_O molecule. In this configuration, the two CH_2_O molecules exhibit distinctly different adsorption behaviors. One molecule follows the same bridge-site adsorption mode as configuration G2, bonding with two coordination-unsaturated Zn atoms at the step edge via its oxygen atom, with Zn–O bond lengths of 2.15 Å and 2.09 Å. The second CH_2_O molecule undergoes spontaneous dissociation into a CHO fragment and a hydrogen atom, corresponding to a typical dissociative adsorption pathway. The released hydrogen atom is captured by a surface oxygen atom, forming a stable O–H bond. The remaining CHO fragment is adsorbed above the center of the hexagonal ring of the ZnO surface, at a vertical distance of 2.61 Å from the surface. The carbon atom is oriented toward a surface Zn atom, while the oxygen atom faces the dissociated hydrogen atom, with a C–H separation of 1.61 Å. Consistent with configuration G2, the CHO fragment is aligned along the [0001] direction perpendicular to the ZnO $\left[20\bar{2}1\right]$ surface. After adsorption, the surface undergoes significant structural relaxation, accompanied by notable changes in local bond angles. In particular, the Zn–O–Zn bond angle relaxes to 110.76°, while the O–Zn–O bond angle adjusts to 96.54°, indicating substantial surface reconstruction induced by dissociative adsorption.

### 3.2. Adsorption Configuration at 0.5 ML Coverage

**Figure 5:**
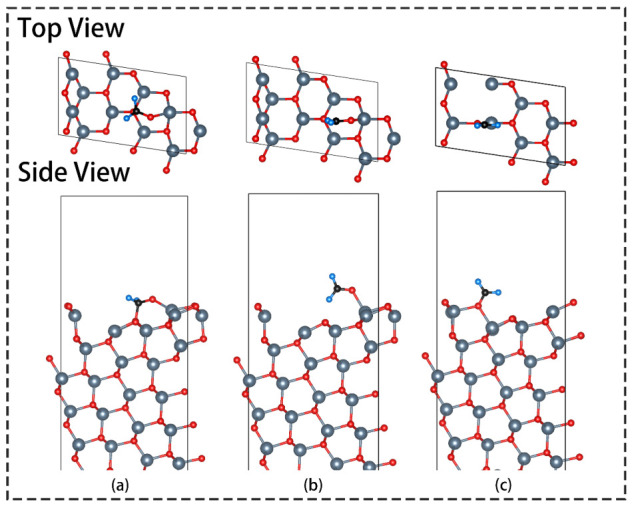
Three adsorption configurations on a 2 × 1 unit cell of the ZnO $\left[20\bar{2}1\right]$ facet at 0.5 ML coverage: (**a**) the J1 configuration, which has the highest adsorption energy; (**b**) the J2 configuration, which involves end-site adsorption; and (**c**) the J3 configuration, in which multiple Zn atoms fill the hanging bonds at the step edges.

To investigate the intrinsic adsorption behavior of CH_2_O molecules on the ZnO $\left[20\bar{2}1\right]$ high-index polar surface at low coverage, a 0.5 ML model was constructed with one CH_2_O molecule adsorbed in a 2 × 1 supercell. After full structural relaxation of all initial adsorption configurations, three energetically stable adsorption structures were obtained. The optimized geometries are shown in Figure 5. Based on adsorption energy (from higher to lower magnitude, corresponding to decreasing adsorption strength and stability), the three configurations are labeled J1, J2, and J3. Among them, configuration J1, shown in Figure 5a, is the most stable structure, with an adsorption energy of −1.35 eV per CH_2_O molecule. This configuration corresponds to a typical bidentate chemisorption mode. Structural analysis indicates that the CH_2_O molecule interacts strongly with the surface through two active sites: the carbon atom forms a C–O bond with a surface coordination-unsaturated oxygen atom, while the terminal oxygen atom forms a Zn–O coordination bond with a surface Zn atom. The corresponding bond lengths are 1.57 Å and 1.92 Å, respectively, both significantly shorter than typical Zn–O bond lengths in bulk ZnO, confirming strong chemisorption. Upon adsorption, the intrinsic sp^2^ hybridization of the CH_2_O molecule is completely disrupted, and the carbon atom adopts a distorted sp^3^-like tetrahedral configuration. Meanwhile, the surface undergoes slight reconstruction, with the O–Zn–O bond angle relaxing to 105.32°. Configuration J2, shown in Figure 5b, has an adsorption energy of −1.34 eV per CH_2_O molecule and corresponds to a monodentate top-site adsorption mode. In this configuration, the CH_2_O molecule interacts primarily through its terminal oxygen atom, which forms a Zn–O bond with a surface Zn atom (bond length: 2.13 Å). Unlike J1, the molecule retains its intrinsic planar sp^2^ geometry without significant structural distortion. In addition, a weak hydrogen-bonding interaction forms between a hydrogen atom of CH_2_O and a surface oxygen atom, resulting in a slight shift in the molecular orientation toward the surface. The substrate also undergoes minor relaxation, with the O–Zn–O bond angle adjusting to 106.2°. Configuration J3 exhibits an adsorption energy of −1.31 eV per CH_2_O molecule and corresponds to a typical bidentate bridge-site adsorption mode. Its adsorption behavior is consistent with the previously discussed G2 and H1 configurations at higher coverages. In this case, the CH_2_O molecule adsorbs at the step-edge active region, with its terminal oxygen atom simultaneously coordinating with two undercoordinated Zn atoms, forming Zn–O bonds of 2.16 Å and 2.10 Å. The molecule is oriented perpendicular to the ZnO $\left[20\bar{2}1\right]$ surface and aligned along the [0001] direction, consistent with the bridge-type adsorption geometry observed in earlier configurations. Importantly, this adsorption process induces negligible surface reconstruction, and the substrate geometry remains largely unchanged, indicating the structural stability and reversibility of this bridge-site adsorption mode.

### 3.3. Electronic Properties

#### 3.3.1. Band Structure

**Figure 6:**
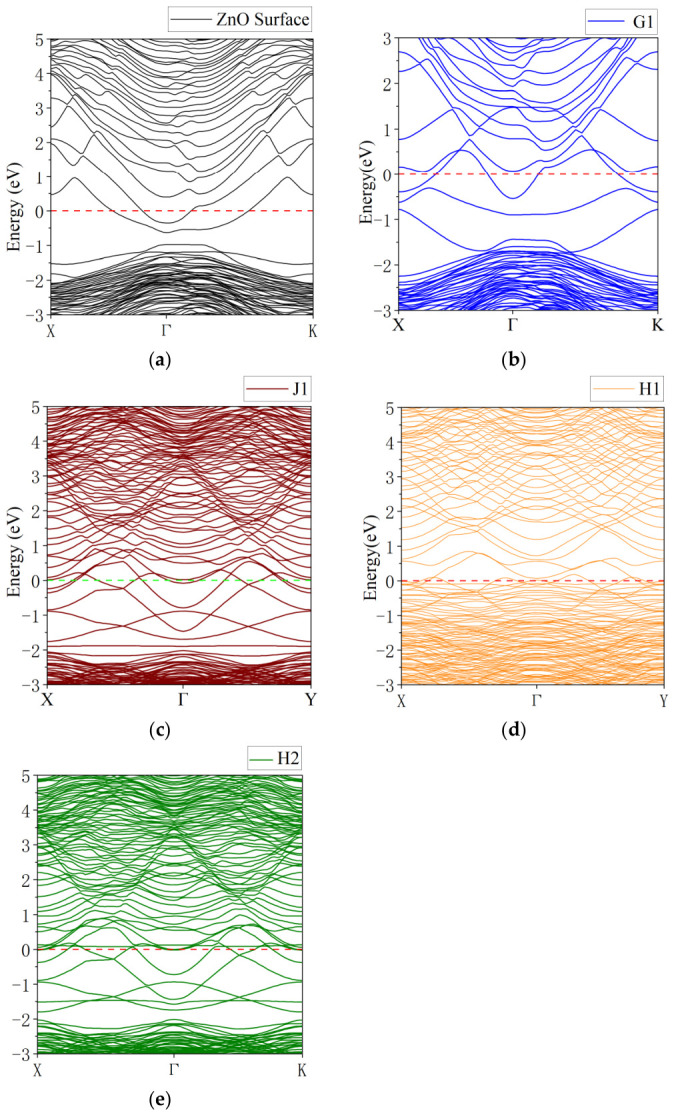
Band structure diagrams of stable configurations on the ZnO [$20\bar{2}1$] surface. (**a**) Band structure of the clean ZnO [$20\bar{2}1$] surface; (**b**) Band structure of configuration G1; (**c**) Band structure of configuration J1; (**d**) Band structure of configuration H1; (**e**) Band structure of configuration H2. The red and green dashed lines in all panels denote the position of the Fermi level.

To elucidate the effect of CH_2_O adsorption, band structure calculations were calculated with the Fermi level aligned to 0 eV (Figure 6). Unlike the intrinsic wide-bandgap semiconducting nature of bulk ZnO, the pristine ZnO [$20\bar{2}1$] surface exhibits distinct metallic behavior, with the Fermi level intersecting multiple continuous bands (Figure 6a). This metallicity, characterized by an effectively closed or negative bandgap, originates from the unique atomic configuration of the high-index polar surface. The abundant step edges and low-coordination atoms introduce a high density of unsaturated dangling bonds, which generate dense surface defect states within the intrinsic bandgap and induce strong orbital hybridization near the Fermi level.

After CH_2_O adsorption forming the globally most stable G1 configuration, the electronic band structure undergoes pronounced reconstruction (Figure 6b). Although the system remains metallic overall, these continuous surface-state bands are distinctly disrupted. In particular, bands near the Fermi level exhibit noticeable bending, and several bands in the energy range from 0 to −1.0 eV show significantly reduced dispersion and tend toward flat-band behavior. This behavior directly indicates strong orbital hybridization and wavefunction overlap between the frontier molecular orbitals of CH_2_O and the intrinsic surface states of ZnO. which is a direct signature of enhanced electronic localization originating from the formation of interfacial Zn–O coordination bonds. As a result, the original continuous surface-state manifold is broken, and these states are reorganized into localized interfacial hybridized bonding states. Although the system does not transition fully into a semiconducting state, the strong orbital hybridization effectively passivates part of the high-density dangling bond states on the surface, driving the electronic structure toward a more energetically stable equilibrium state.

In the band structure of the most stable J1 configuration at 0.5 ML coverage (Figure 6c), several bands still cross the Fermi level, indicating that the system retains residual metallic characteristics at the macroscopic level. However, the distribution of electronic states near the Fermi level undergoes substantial reconstruction. Near the Brillouin zone center, a pronounced band repulsion effect emerges, where valence bands near the Fermi level are pushed downward below −0.5 eV and conduction bands are shifted upward, resulting in the formation of a clear pseudogap. Although the DOS at the Fermi level is significantly reduced, it does not completely vanish. This behavior indicates that originally delocalized and highly active surface electrons are effectively passivated due to the formation of dual C–O and Zn–O chemical bonds between CH_2_O and the ZnO surface, which partially suppresses conduction channels in this momentum region. Multiple bands near the Fermi level exhibit pronounced splitting and anti-crossing features, indicating strong orbital hybridization between surface states and CH_2_O molecular orbitals, leading to the formation of extended interfacial electronic states. Furthermore, bands below the Fermi level show markedly reduced dispersion and become nearly flat, corresponding to a significant increase in carrier effective mass directly reflecting strong electron localization induced by adsorption.

In the band structure of the associative H1 configuration at 1 ML coverage (double-molecule dimerization bridge-site adsorption) (Figure 6d), a small direct bandgap of 0.04 eV opens at the Fermi level after CH_2_O adsorption. This completely suppresses the metallic nature of the pristine surface, driving a transition to a narrow-bandgap semiconducting state. This band evolution originates from the synergistic adsorption of two CH_2_O molecules. During adsorption, the molecules not only form strong Zn–O coordination bonds with surface Zn atoms via their terminal oxygen atoms, but also undergo C–C covalent coupling between the carbon atoms, leading to dimer formation. To form a stable C–C single bond, the hybridization of the carbon atoms changes from planar sp^2^ to tetrahedral sp^3^, indicating a fundamental reconstruction of the molecular electronic structure. This transformation consumes electrons that were originally part of highly delocalized states near the Fermi level, transferring them into localized bonding states. Meanwhile, strong orbital interactions induce pronounced energy-level repulsion that pushes the corresponding antibonding states upward into the conduction band region. The combined effect of stabilizing bonding states and destabilizing antibonding states effectively removes the continuous metallic states that previously crossed the Fermi level, significantly passivating defect states associated with unsaturated surface dangling bonds. Consequently, a finite bandgap of 0.04 eV emerges at the Fermi level, marking a metal-to-narrow-gap semiconductor transition induced by cooperative molecular adsorption.

In the band structure of the dissociative H2 configuration at 1 ML coverage (Figure 6e), the metallic surface states that densely cross the Fermi level in the pristine surface are completely eliminated. This results from the effective passivation of a large number of unsaturated dangling bonds on the polar surface by hydrogen atoms and CHO fragments generated through CH_2_O dissociation, significantly stabilizing the high-energy surface states and shifting them downward into deeper valence-band regions. This electronic reconstruction leads to a sharp reduction in the DOS at the Fermi level and the formation of a pronounced pseudogap along most high-symmetry paths in the Brillouin zone. The system therefore exhibits a strong tendency toward recovery of the intrinsic semiconducting band characteristics of ZnO. In addition, numerous highly localized and nearly dispersionless bands emerge in the energy range below −1.51 eV within the valence band, corresponding to the formation of strong O–H covalent bonds generated by CH_2_O dissociation, as well as robust coordination interactions between CHO fragments and surface atoms. These localized electronic states provide a direct fingerprint of strong chemical bonding and surface passivation in the dissociative adsorption configuration.

#### 3.3.2. Partial DOS (PDOS) and Charge Density Difference

Previous band structure analysis has confirmed that CH_2_O adsorption on the ZnO [$20\bar{2}1$] high-index polar surface induces significant reconstruction of the electronic structure, providing direct evidence of strong chemisorption between the adsorbate and the substrate. To further elucidate the microscopic origin of this strong interaction from the perspective of atomic orbitals, this section systematically investigates orbital hybridization, charge transfer, and interfacial bonding mechanisms between CH_2_O molecules and the ZnO substrate through PDOS analysis. In addition, CDD calculations are employed to visually characterize and quantitatively evaluate the interfacial charge redistribution behavior during the adsorption process.

**Figure 7:**
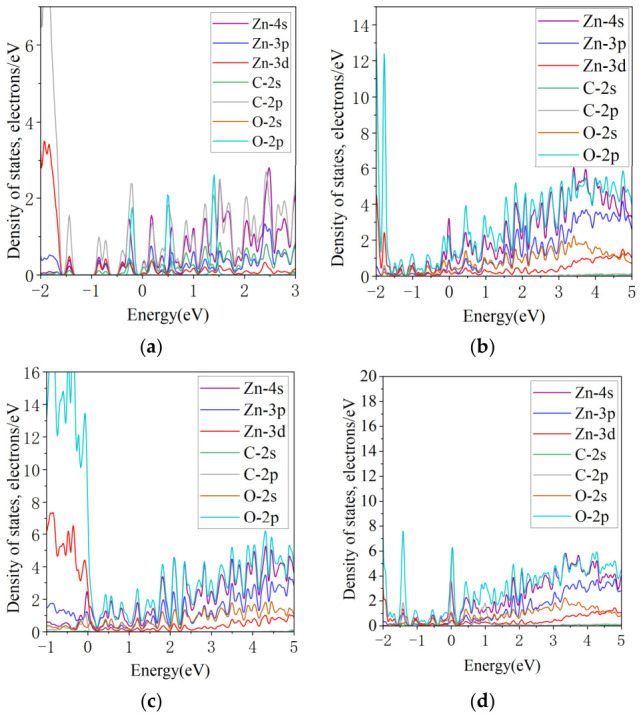
PDOS plots of four representative configurations. (**a**) PDOS plot of configuration G1; (**b**) PDOS plot of configuration J1; (**c**,**d**) are PDOS plots of configurations H1 and H2 under 1 ML coverage in a 2 × 1 supercell.

**Figure 8:**
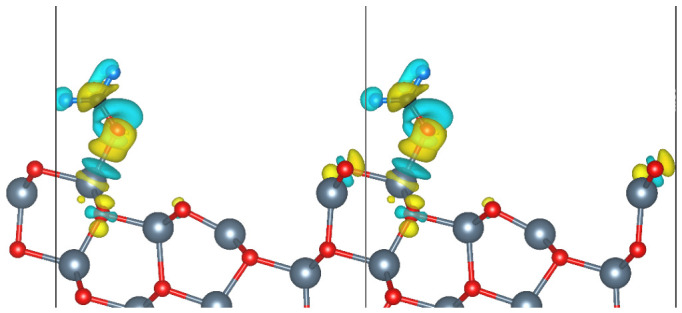
Differential charge density profile for configuration G1. The yellow regions represent charge accumulation areas, and the blue regions represent charge depletion areas. The same color coding is used for all subsequent charge density plots.

In the PDOS of the G1 configuration (Figure 7a), the deep valence band region (−2.0 eV to −1.55 eV) is primarily dominated by hybrid contributions of substrate Zn-3d orbitals and molecular C-2p orbitals, where localized Zn-3d resonance peaks and corresponding C-2p bonding states confirm significant adsorbate-substrate electronic interaction. Near the Fermi level, multiple sharp and discrete peaks emerge as characteristic signatures of strong chemisorption, exhibiting significant overlap between molecular O-2p/C-2p orbitals and unsaturated surface Zn-4s/3p orbitals at several energy levels (e.g., −0.82 eV, −0.24 eV, and 0.5 eV). These resonance features indicate strong orbital hybridization between the frontier p orbitals of CH_2_O and the unsaturated s/p orbitals of surface Zn atoms, confirming the formation of robust Zn–O interfacial coordination bonds that explain the high adsorption stability observed in the structural analysis. In the high-energy conduction band region (1.0–3.0 eV), hybridized states contributed by Zn-4s, C-2p, and O-2p orbitals emerge from the mixing of CH_2_O antibonding orbitals with empty ZnO surface states, disrupting the previously continuous delocalized surface-state distribution. As a result, discrete electronic transition channels are introduced, which are directly relevant to the thermal excitation-driven resistance response of gas-sensing behavior. In the CDD profile (Figure 8), a continuous and intense charge accumulation region at the interface between the CH_2_O oxygen atom and the surface Zn atom provides direct real-space evidence for Zn–O covalent coordination bond formation, fully consistent with the PDOS results. Meanwhile, a distinct charge depletion region appears around the bonded Zn atom, indicating significant electron transfer toward the bonding interface, further confirming the strong chemisorption and substantial interfacial charge redistribution.

**Figure 9:**
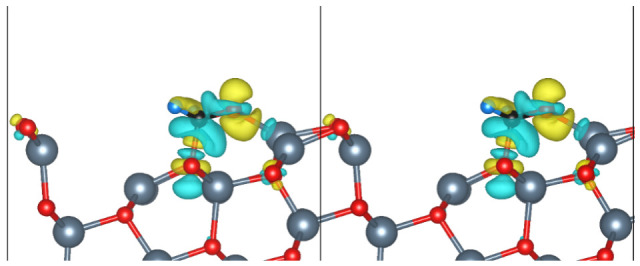
Differential charge density for configuration J1.

In the PDOS of the J1 configuration (Figure 7b), an intense and sharply defined resonance peak is observed in the total DOS in the deep valence band region around −2.0 eV, primarily contributed by Zn-3d orbitals of the ZnO substrate and O-2p orbitals. This indicates that the adsorption process not only modifies surface electronic states but also induces a redistribution of deeper lattice bonding electrons, while the overall ZnO lattice framework remains structurally stable. Near the Fermi level, the continuous delocalized surface states present in the pristine surface are completely disrupted, giving rise to a series of strongly hybridized, narrow, and highly localized peaks. Such sharp features correspond to a significant increase in carrier effective mass, which is fully consistent with the flat-band characteristics and strong electronic localization observed in the band structure analysis. Strong orbital overlap between the O-2p orbitals of the CH_2_O molecule and the Zn-4s and Zn-3p orbitals of surface Zn atoms emerges at energies near 0 eV, 0.5 eV, and 0.8 eV, confirming that, following the distortion of the CH_2_O carbon atom from sp^2^ toward an sp^3^-like hybridization state, the frontier orbitals of CH_2_O at both active sites undergo strong covalent interaction with coordination-unsaturated surface atoms, forming a stable bidentate coordination configuration. In the CDD profile (Figure 9), charge redistribution is strongly localized at the two bonding interfaces between CH_2_O and the ZnO surface: one between the CH_2_O carbon atom and a surface oxygen atom, and the other between the CH_2_O oxygen atom and a surface zinc atom. In both regions, pronounced charge accumulation is observed, providing direct real-space evidence for the formation of C–O covalent bonding and Zn–O coordination bonding, fully consistent with the PDOS analysis. Simultaneously, a distinct charge depletion region surrounds the original C=O bond of CH_2_O, indicating that the π-bonding electrons are significantly depleted and transferred toward the interfacial bonding regions. This electron redistribution leads to the breaking of the original C=O double bond, driving structural distortion of the molecule and facilitating the transition of the carbon atom sp2 toward an sp3-like hybridization state. Surface Zn and O atoms involved in bonding also exhibit clear charge depletion, confirming that both substrate atoms and the CH_2_O molecule jointly contribute valence electrons to the newly formed interfacial bonds, further validating the strong chemisorption nature of the bidentate adsorption configuration.

**Figure 10:**
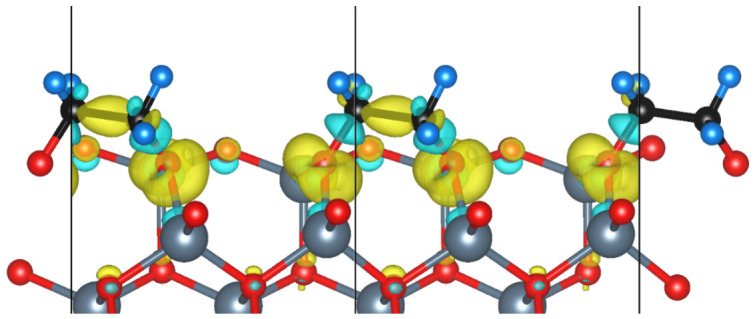
Differential charge density plot for configuration H1.

In the PDOS of the H1 configuration (Figure 7c), the C-2p orbitals of CH_2_O exhibit multiple sharp and strongly localized peaks in the upper valence band region below the Fermi level (−2.0 eV to 0 eV). Notably, a pronounced resonance overlap is observed between the C-2p states of the two CH_2_O molecules, which directly indicates strong inter-molecular orbital coupling, providing clear electronic-structure evidence for the formation of a stable C–C covalent bond that confirms the dimerization mechanism and explains the stability of this adsorption configuration. Simultaneously, strong hybridization is observed between the O-2p orbitals of CH_2_O and the Zn-4s and Zn-3p orbitals of surface Zn atoms, with two well-aligned resonance peaks appearing at approximately 0.62 eV and 0.84 eV. This strong orbital overlap indicates that the lone-pair electrons of CH_2_O oxygen atoms form robust coordination interactions with the unsaturated orbitals of surface Zn atoms, fully consistent with the bridge-site adsorption geometry identified in the structural analysis. In the CDD profile (Figure 10), charge accumulation is primarily localized in two regions: the C–C bond region between the two CH_2_O molecules, and the interfacial region between CH_2_O oxygen atoms and surface Zn atoms. The former provides direct real-space confirmation of C–C bond formation, consistent with the PDOS evidence of C-2p orbital resonance, while the latter verifies the formation of Zn–O bridge-type coordination bonds. In contrast, significant charge depletion is observed in the original C=O bond region of CH_2_O as well as around surface Zn atoms involved in bonding, clearly indicating that the original C=O π-bond is completely disrupted, with valence electrons being transferred into newly formed C–C covalent and Zn–O coordination bonds. This process drives the carbon atoms toward an sp^3^-like hybridization state while simultaneously passivating high-energy dangling bond states on the ZnO surface, resulting in a pronounced electronic reconstruction consistent with the previously observed transition from metallic behavior to a narrow-bandgap semiconducting state in the band structure analysis.

**Figure 11:**
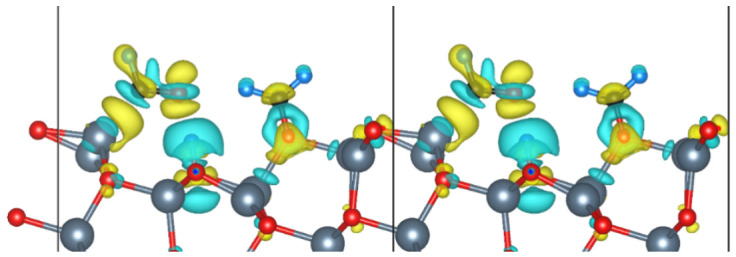
Differential charge density plot for configuration H2.

In the PDOS of the H2 configuration (Figure 7d), the most prominent feature is a strong hybridization peak at the Fermi level, jointly contributed by O-2p, Zn-4s, and C-2p orbitals. This behavior is directly associated with the spontaneous dissociative adsorption of CH_2_O at surface active sites, where the detached hydrogen atom binds with a surface lattice oxygen atom to form a hydroxyl group, while the remaining aldehyde fragment forms a strong coordination bond with a surface Zn atom. This redox process releases additional electrons into the ZnO conduction band, resulting in an upward shift in the Fermi level and its intersection with conduction band states, ultimately giving rise to the pronounced hybridization peak at the Fermi level that confirms a strong n-type doping effect on the ZnO surface, which is directly related to the conductivity change mechanism in gas sensors. In the valence band region, the C-2p and O-2p orbitals of the aldehyde fragment and hydroxyl group exhibit multiple sharp and localized resonance peaks with Zn-3d and O-2p orbitals of the substrate, corresponding to newly formed stable chemical bonding states (including O–H bonds and Zn–O coordination bonds) that explain the energetic stability of the dissociative adsorption configuration. In the CDD profile (Figure 11), a pronounced charge depletion region around the original C–H bond of CH_2_O directly confirms bond cleavage and molecular dissociation under the catalytic action of surface active sites. Meanwhile, strong charge accumulation is found between the dissociated hydrogen atom and a low-coordination surface oxygen atom, confirming the formation of an O–H covalent bond, while significant charge accumulation at the interface between the aldehyde fragment and surface Zn atoms indicates the formation of strong Zn–O coordination bonds. In addition, partial charge accumulation extends around surface Zn atoms, suggesting that electrons released from the dissociation process are transferred to the ZnO substrate, increasing the carrier concentration of the system in full consistency with the PDOS analysis and confirming the n-type doping effect induced by CH_2_O dissociation.

## 4. Conclusions

This study pioneers the application of the ZnO [$20\bar{2}1$] high-index polar surface for formaldehyde detection, employing first-principles calculations based on DFT to systematically investigate its adsorption behavior and electronic structure evolution. By focusing on this specific high-index facet for the first time, we reveal the profound impact of surface structural complexity on gas sensing performance. The main conclusions are as follows. First, breaking away from the limitations of traditional low-index surfaces, the pristine ZnO [$20\bar{2}1$] high-index surface exhibits intrinsic metallic behavior driven by a uniquely high density of coordination-unsaturated atoms and stepped defect states. These high-index structural features provide an unprecedented abundance of high-activity sites for CH_2_O adsorption, constituting the fundamental origin of its superior gas-sensing performance. During single-molecule adsorption, CH_2_O undergoes strong chemisorption on the surface, with significant hybridization between frontier molecular orbitals and surface states, leading to the formation of stable interfacial bonds. This process also induces pronounced electronic localization and effectively passivates high-energy surface dangling bonds. At higher coverage, CH_2_O exhibits two distinct adsorption pathways. In the associative dimerization mode, adsorption drives a transition of the surface electronic structure from metallic behavior to a narrow-bandgap semiconductor state (bandgap of approximately 0.04 eV). In contrast, spontaneous dissociative adsorption injects a substantial number of free electrons into the ZnO substrate, resulting in strong n-type metallization of the surface. These two competing pathways provide a unified microscopic explanation for the experimentally observed macroscopic phenomenon in which CH_2_O adsorption induces a sharp decrease in the resistivity of ZnO-based sensing materials. Overall, this work establishes the critical role of high-index facet engineering and clarifies the structure–property relationship governing CH_2_O adsorption on the ZnO [$20\bar{2}1$] surface, providing a robust theoretical breakthrough for the rational design of high-performance formaldehyde gas sensors.

## Data Availability

The data that support the findings of this study are available from the corresponding author upon reasonable request. This includes the structural models, calculation input/output files, and adsorption energy results used in this work.
